# Underwater object detection method based on learnable query recall mechanism and lightweight adapter

**DOI:** 10.1371/journal.pone.0298739

**Published:** 2024-02-28

**Authors:** Xi Lin, Xixia Huang, Le Wang

**Affiliations:** Institute of Logistics Science and Engineering, Shanghai Maritime University, Shanghai, People’s Republic of China; Vellore Institute of Technology: VIT University, INDIA

## Abstract

With the rapid development of ocean observation technology, underwater object detection has begun to occupy an essential position in the fields of aquaculture, environmental monitoring, marine science, etc. However, due to the problems unique to underwater images such as severe noise, blurred objects, and multi-scale, deep learning-based target detection algorithms lack sufficient capabilities to cope with these challenges. To address these issues, we improve DETR to make it well suited for underwater scenarios. First, a simple and effective learnable query recall mechanism is proposed to mitigate the effect of noise and can significantly improve the detection performance of the object. Second, for underwater small and irregular object detection, a lightweight adapter is designed to provide multi-scale features for the encoding and decoding stages. Third, the regression mechanism of the bounding box is optimized using the combination loss of smooth *L*_1_ and CIoU. Finally, we validate the designed network against other state-of-the-art methods on the RUOD dataset. The experimental results show that the proposed method is effective.

## 1. Introduction

In recent years, due to the rapid development of sensor technology and image processing algorithms, underwater object detection technology has been more and more widely used, such as fishery farming, underwater rescue, marine resources development and other fields [1]. Traditional underwater object localization and recognition usually need to be done manually, which is not only inefficient, but also has high false detection rate and high leakage rate [2]. Therefore, underwater object detection has become one of the most challenging tasks in current computer vision technology.

Underwater optical image detection faces a lot of challenges compared to generalized detection scenarios [3]. First, underwater optical image datasets are inadequate. The complex underwater ecosystem places high demands on underwater imaging equipment, making it difficult to acquire abundant and massive underwater images. Second, underwater objects are prone to masking as well as overlapping due to factors such as their habits. Moreover, marine organisms are characterized by low interclass variance and high intraclass variance. For example, plastic bags and jellyfish are different object types, but they are relatively similar in appearance. And while carp and sharks are both fish, they differ greatly in appearance. More importantly, underwater optical image detection also faces unique challenges in underwater scenes [4], it can be divided into three main points:

1. Underwater images are usually heavily noisy. In underwater scenes, relatively large noise signals are generated due to the absorption and scattering effects of light by the medium in the water, resulting in reduced image contrast, color distortion and image blurring. This noise signal increases the challenge of inter-class similarity, leading to confusion between object classes and background classes.
2. There are more types of small underwater objects, such as fish, starfish, sea urchins and scallops. Small objects have limited features available, and as the network deepens, their feature information may disappear completely, making them susceptible to missed detection.
3. The multi-scale problem of underwater objects is obvious, which is characterized by irregular dimensions, extreme aspect ratios, and variable shapes, and is prone to misdetection.

Under the combined effect of the above factors, it is difficult for the current generalized target detection methods to achieve better detection results for underwater objects. Thus, in order to solve these problems, a DETR-based underwater object detection network is designed in this paper. In the design process, a learnable query recall mechanism is proposed for identifying and localizing objects to speed up network convergence. In the feature extraction part, a lightweight adapter is designed. In the loss function part, the combination of smooth *L*_1_ [5] loss and CIoU [6] loss is used for the regression loss of the bounding box.

In summary, our contributions mainly include the following aspects:

1. In order to solve the problem of severe underwater image noise and blurred images leading to inconspicuous object features, we propose a learnable query recall mechanism. This mechanism can effectively introduce early queries into the later stages and reduce the influence of noise signals, thus improving the underwater object detection accuracy.
2. In order to solve the small-object, multi-scale problem for underwater objects, we design a lightweight adapter. This module can effectively extract and fuse multi-scale feature information with only a small increase in the number of model parameters.
3. In order to localize the prediction bounding box more accurately, we use the combination of smooth *L*_1_ loss and CIoU loss for the localization loss of the bounding box.

The remainder of the paper is structured in the following way. In Section 2, we outline the related work, which mainly includes generic object detection and underwater object detection. In Section 3, we focus on our method, including the core network structure and loss function. In Section 4, we describe the experimental details and analyze the results of the experiment. In Section 5, we give a brief conclusion to our work in this paper.

## 2. Related work

### 2.1 Generic object detection

Currently the mainstream general-purpose object detection algorithms are based on deep learning, and the detection algorithms can be roughly classified into three categories according to the workflow: anchor-based methods, anchor-free methods, and transformer-based methods [7]. Both anchor-based and anchor-free methods are usually CNN-based detectors [8]. Anchor-based methods can be divided into two-stage methods and one-stage methods. Two-stage methods, represented by Faster R-CNN [9], Mask R-CNN [10] and Cascade R-CNN [11], divide the detection task into two phases: extracting candidate regions and classifying and locating them. By finding out where the target object appears first, the candidate frames are initially obtained followed by exact position regression and classification of objects. Two-stage methods have the advantage of higher detection accuracy and the disadvantage that they are usually slower. One-stage methods are represented by SSD [12], RetinaNet [13], and YOLO [14]. Considering object detection as a regression problem, instead of extracting candidate regions first, neural networks are used to detect and locate objects from the whole image, directly regressing category probabilities and position coordinate values. One-stage methods have the advantage of faster detection and can be applied to real-time detection scenarios.

Anchor-free methods can be classified into key-point and center-point methods, represented by CenterNet [15], FCOS [16], etc. CenterNet predicts the location of several key points in the bounding box by predicting the location of the key points and decoding the key points into a prediction box. FCOS encodes the ground truth as an anchor point with the distance from the corresponding point to the boundary, where an anchor point is a pixel on the feature pyramid map whose position is associated with the feature. The essential idea of the anchor-free methods is to discard the manually designed anchors and to determine the positive and negative samples in a more streamlined way by means of key-point or center-point [17]. This further streamlines the process of object detection, which reduces the design of relevant hyperparameters and makes model building easier.

With the popularity of transformer in the field of computer vision, the Facebook researchers cleverly used the transformer architecture to propose a novel object detector DETR [18]. It relies on a multi-stage transformer encoder and decoder layer that updates learnable queries into object features that can be decoded into bounding box predictions at each stage. During the training process, it uses bipartite graph matching to dynamically determine the positive and negative samples [19]. DETR simplifies the detection framework by converting the object detection task into an ensemble prediction task for the first time, without the need for pre-designed candidate frames with prior knowledge or NMS post-processing procedures [20], thus enabling end-to-end object detection. However, DETR usually exists problems such as long training period and slow convergence speed, while there is still a lot of room for improvement in the detection ability of small objects [21].

These object detection algorithms are usually trained and evaluated on existing large-scale generalized datasets such as PASCAL VOC [22] and MS COCO [23]. Despite the success of these object detection algorithms on generalized detection scenarios, they still face challenges when dealing with underwater scenarios.

### 2.2 Underwater object detection

Underwater optical images usually suffer from severe noise, image blurring, low contrast, color distortion, etc., which makes generic object detection algorithms to deal with underwater images directly often not effective [24]. In order to solve the problem of underwater image blurring, Lin et al. [25] proposed a RoIMix image enhancement method that performs a proposal-level fusion between multiple images to generate different training samples. RoIMix is designed to simulate underwater blurred, overlapping, and occluded objects, so that the model implicitly learns the ability to detect underwater targets. This method improves the performance of underwater object detection, but it is designed based on the Faster R-CNN and its variants, and the algorithm lacks certain generality. Aiming at underwater image blurring and severe noise interference, Chen et al. [26] proposed a sample-weighted hyper network for generating multiple high-resolution, semantically-rich feature maps to improve the detection accuracy of small objects. The noise robust training paradigm is also used to solve the noise problem encountered in underwater object detection by first learning clean underwater data and then learning different noisy data. Although the network can effectively solve problems such as image noise and small objects, the computational complexity is much higher than that of the currently popular non-integrated models because it is a deeply integrated model.

To address the problem of underwater small objects and multiple scales, Wang et al. [27] improved the Faster R-CNN by using Res2Net101 [28] as a feature extraction network to enhance the expression of sensory fields at each network layer, and improved the bounding box regression mechanism using GIoU [29] and Soft-NMS [30]. This method effectively improves the underwater detection performance, but the CNN is still the backbone network of the detector and it is difficult to extract the global representation of the object in a robust manner. Liu et al. [31] proposed a two-stage network based on Swin Transformer [32], which superimposes and fuses images of different resolutions by adding a path aggregation network to reduce the problem of missed and false detection of objects of different sizes underwater. But the method has a relatively large model size and is not fast to detect. Chen et al. [33] proposed a lightweight transformer network capable of extracting global contextual information, using a fine-grained feature pyramid network to achieve efficient detection of small underwater objects. This model is not ideal in terms of the actual operating speed of the device, despite achieving relatively small results in terms of the number of parameters. In order to strike a balance between detection performance and model lightweighting, Liu et al. [34] embedded the transformer encoder and coordinate attention module in YOLOV5 to improve the detection performance of underwater objects, but the model is too complex and needs a lot of improvement work to realize practical applications. Lei et al. [35] used Swin Transformer as the backbone network of YOLOV5 to make the model suitable for detecting underwater fuzzy objects. The confidence loss function was also improved to bias the network towards learning high-quality positive anchor boxes to enhance the network’s ability to detect objects. Tang et al. [36] proposed a hybrid DETR-YOLO detection model, which utilizes the DETR module for global feature extraction of the input and combines the lightweight advantages of YOLO to improve the accuracy of small object detection. However, the model is mainly applied to side-scan sonar and cannot be directly migrated to underwater optical images.

## 3. Method

The model in this paper is based on DETR, which is one of the most advanced object detection algorithms available. DETR converts the problem of object detection into one of ensemble prediction by enforcing the determination of unique predictions through bipartite graph matching, effectively eliminating many of the manually pre-set components, such as candidate box generation and non-maximal value suppression. We follow DETR with transformer encoder and decoder structures. The input image is passed through the backbone network to extract features. The encoder performs global modeling of image features to establish remote dependencies. The decoder takes the object queries and the image features obtained by the encoder and decodes them and sends them to the prediction feed-forward networks to get the output. In this section, the network structure, the query recall mechanism, the lightweight adapter, and the localization loss are described in detail.

### 3.1 Network architecture

**Fig 1:**
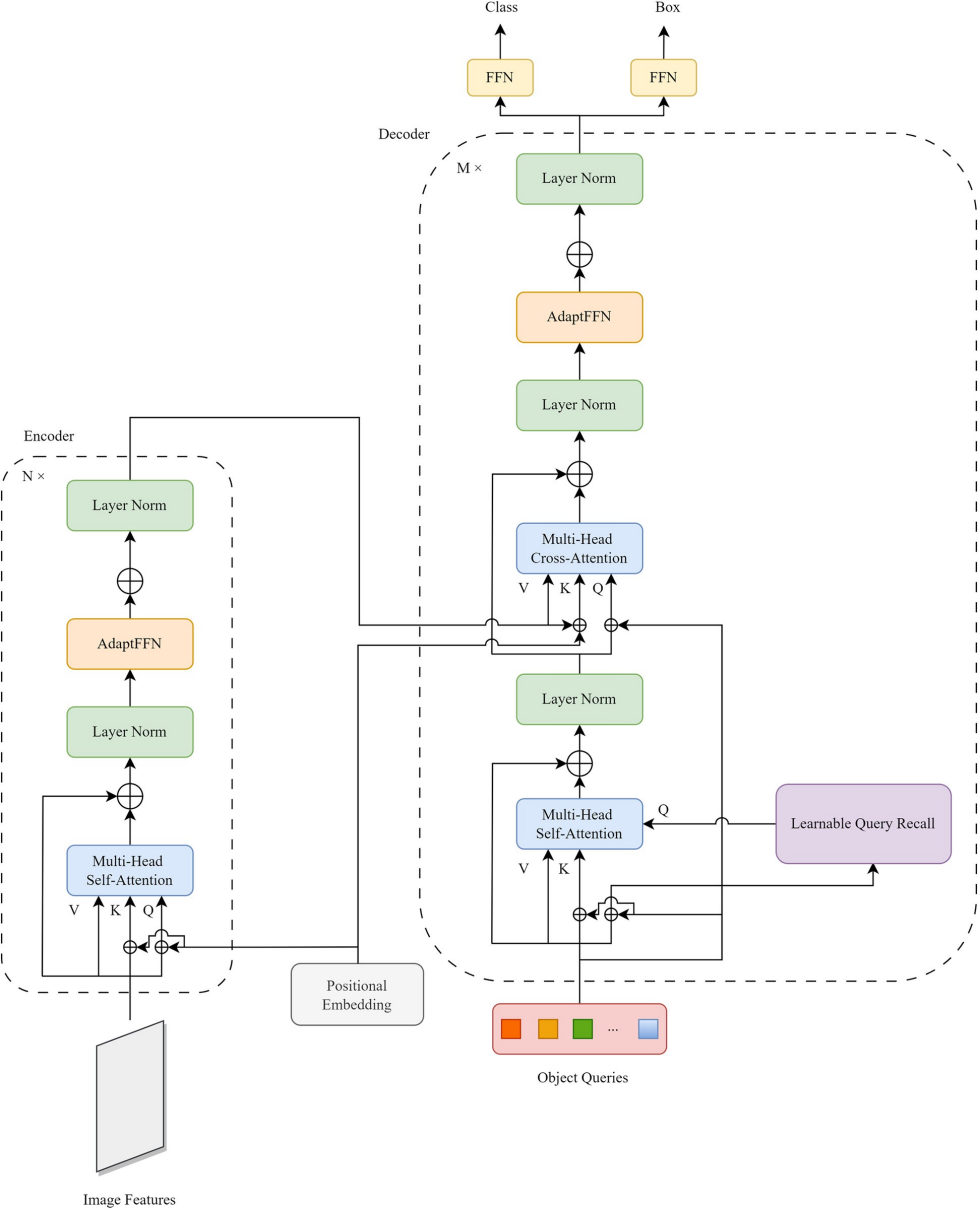
Architecture of network’s transformer.

As shown in Fig 1, considering the requirements of practical application scenarios for underwater object detection, we redesign the Transformer part for DETR, and retain the default settings for the backbone, i.e., ResNet50 [37] is used for the extraction of compact feature representations. Overall, our proposed model uses a conventional CNN backbone to learn the features of the input image. And the model supplements it with a positional embedding before passing it into a transformer encoder. A transformer decoder then takes object queries as input, and additionally attends them to the encoder output. Finally, we pass each output embedding of the decoder to the feed-forward networks that predict the object’s class and bounding box. In particular, we redesign the architecture of the transformer for the standard DETR model. A learnable query recall mechanism is incorporated into the input of each decoder’s multi-headed self-attentive module query to mitigate the effects of noise and accelerate network convergence. In addition, a lightweight adapter module is incorporated into each encoder and decoder to obtain a deep feature map that incorporates multiple layers of semantic information, making the network robust in extracting underwater object features. The details of these methods will be described in the following sections.

### 3.2 Learnable query recall mechanism

**Fig 2:**
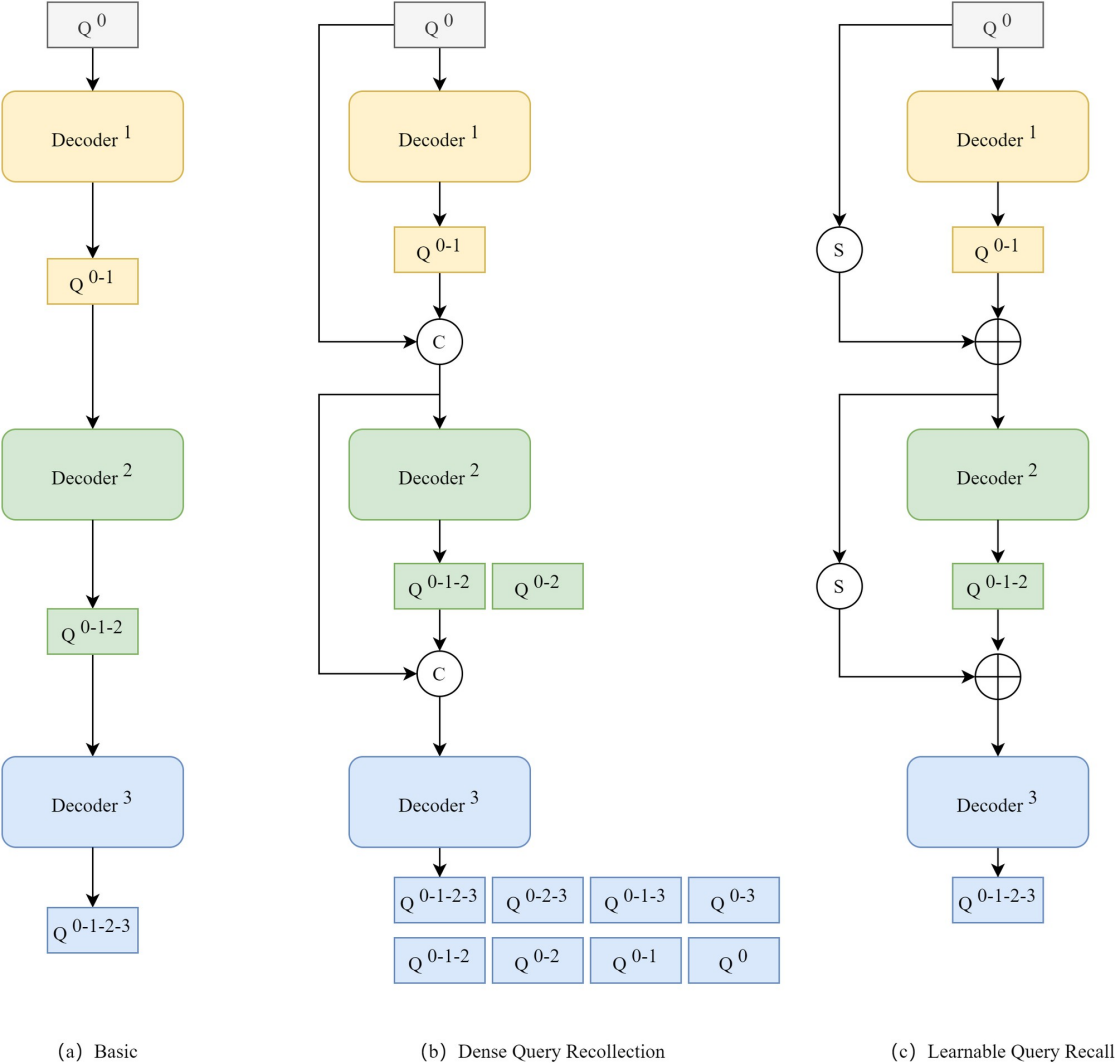
Different query decoding mechanisms. (a) Basic, (b) Dense Query Recollection, (c) Learnable Query Recall.

Due to the absorption and scattering of light by the medium and suspended particles in the water, underwater optical images are usually characterized by severe noise interference, which visually makes the images blurred and visibility reduced. And during the model training process, this problem may result in the texture detail features of the image being difficult to be accurately captured or even lost. Therefore, reasonable elimination of noise interference is essential. The original DETR takes the learnable query and optimizes it through multiple layers of decoders, culminating in the mapping of the prediction header into classification scores and bounding boxes. Since the decoder is sequentially structured and the queries are cascaded, a query at the previous level cannot have an effect on a query at a later level, which results in the presence of cascading errors, where the noise cannot be completely eliminated and may continue to be passed along with the cascading structure. Thus, Chen et al. [38] proposed dense query recollection mechanism that collects queries for each decoding stage and passes them along the original path. This strategy achieves noise mitigation by increasing the number of queries from different phases that are repeatedly supervised. However, this method of intensive query collection is particularly memory intensive and also additionally introduces noise signals that are too early. Therefore, in order to get stable and accurate query during training, we propose a simple and effective learnable query recall mechanism. For each decoder’s multi-head self-attention module query, the previous stage’s query is introduced directly into that stage together as query input. This mechanism can apply early supervisory signals to the decoding phase, mitigating the influence of noise for better performance. As shown in Fig 2, we compare and analyze the proposed learnable query recall mechanism with the original DETR’s query iteration mechanism and the dense query recollection mechanism.

In Fig 2(A), We simplify the query along the basic pathway. The decoding process for the original DETR can be expressed as:

$$Q^{0-i}=D^{i}(Q^{0})$$ (1)

*Q*^0^ is a set of queries that is initialized. *D*^*i*^ is a decoding stage where *i* is stage index. *Q*^0−*i*^ means the final query that *Q*^0^ goes through *D*^*i*^. For the original DETR, queries are updated successively. Taking a 3-stages decoder as an example, we denote *Q*^0−1−2−3^ as the final query that goes through all stages.


$$Q^{0-1-2-3}=D^{3}(D^{2}(D^{1}(Q^{0})))$$ (2)

During training, the queries from each stage, i.e., *Q*^0−1^, *Q*^0−1−2^ and *Q*^0−1−2−3^ are independently followed by Hungarian Assignment that matches ground-truth in a one-to-one assignment, and then followed by loss calculation for supervision.

In Fig 2(B), we densely collect every intermediate query and independently concatenate them to every downstream stage. After each stage, the number of queries grows exponentially, such as (1,2,4,8,16). This intensive collection of each intermediate query, while significantly increasing the number of queries, takes up huge computational resources during training. In addition, if we input an early query that skips too many stages to a far-away late stage, the potential benefit could be overshadowed by the huge learning gap between the stages and query. Thus, we tend to collect intermediate query selectively rather than densely collect all of them.

In Fig 2(C), we selectively collect queries along the basic path, i.e., when moving to the next stage of the decoder, we weight and sum the previous stage’s query with the output query of the previous stage’s decoder to obtain the final input query. Taking a 2-stages decoder as an example, the learnable query recall can be formulated as:

$$Q^{0-1-2}=D^{2}(\left(\alpha \cdot Q^{0})+({\beta \cdot Q}^{0-1}\right))$$ (3)

*α* and *β* are learnable parameters that are automatically updated during training. To ensure optimal performance, we initialize *α* to 1 and *β* to 0. The learnable query recall mechanism reduces the computational burden on the one hand, and on the other hand effectively incorporates contextual information into the query, avoiding the noise impact caused by introducing queries that skip too many stages. In addition, the proposed learnable query recall mechanism mitigates the overfitting problem of the extended network from the perspective of compact representation by introducing two dynamic tuning parameters, *α* and *β*, to keep the increase of model parameters to a minimum.

### 3.3 AdaptFFN

**Fig 3:**
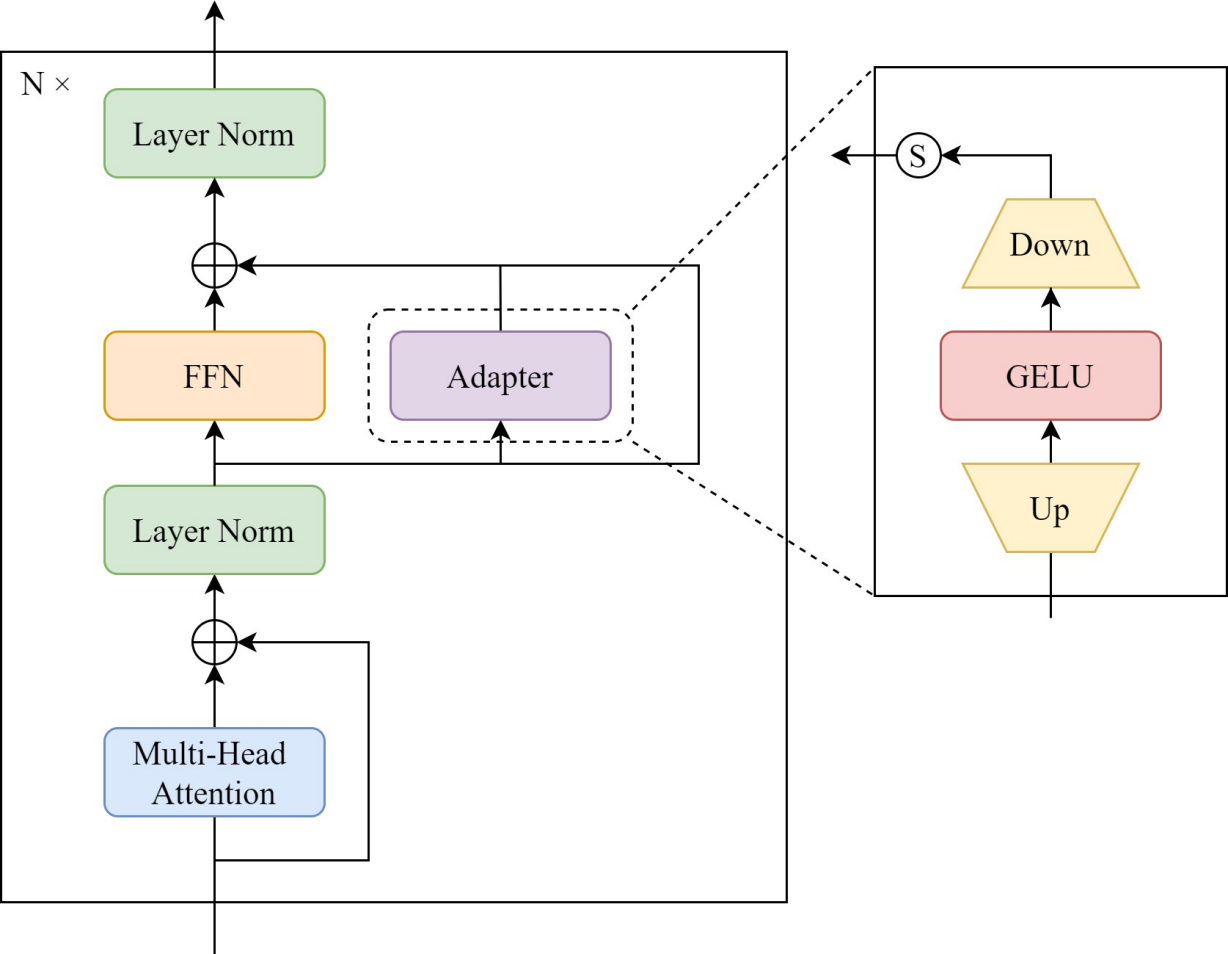
Architecture of AdaptFFN.

Since underwater objects are usually small and of varying scales, direct detection using a generic object detector often results in fine-grained information gradually disappearing during this operation due to the repeated stacking of encoder and decoder modules, where the global context information undergoes multiple up-sampling and down-sampling operations. The obsession with adding feature pyramid structures to the network to extract and fuse features for performance enhancement usually makes the model too large, computationally complex and difficult to deploy in practice. Therefore, in order to make the network suitable for underwater small and irregular object detection scenarios, inspired by the fact that adapter-based fine-tuning performs well in the Natural Language Processing [39–41], we propose a plug-and-play module, namely AdaptFFN. The design principle of AdaptFFN is simply yet effective, which is illustrated in Fig 3. We replaced the feed-forward network (FFN) in the transformer encoder and decoder with AdaptFFN. It is composed of three sub-branches. The left branch has the same FFN as the original network, the middle branch is an additional lightweight module introduced, and the right branch is a residual connection that preserves the inputs. Specifically, the intermediate branch is designed to limit the number of parameters to a structure that includes an upper projection layer with parameter $F_{up}\in R^{D\times \hat{D}}$ and a lower projection layer with parameter $F_{down}\in R^{\hat{D}\times D}$, where $\hat{D}$ is the middle dimension. In addition, there is a GELU layer between these projection layers for non-linear transformation [42]. This module is connected to the FFN structure of the original network (left branch) via a learnable scale factor *s*∈ℝ^*D*^, which is initialized with 0. By designing in parallel, specific features produced by the adapter module can complement those produced by the fixed branch. When it produces a feature channel that is not related to the object to be detected, the scale factor can be adjusted adaptively to realize the suppression effect. It can effectively inhibit the feature channels that are irrelevant to the object to be detected and improve the semantic information of the feature map, which in turn improves the detection accuracy of the object. The process of this module can be formulated as:

$${\hat{x}}_{in}={s\cdot F}_{down}(GELU(F_{up}(x_{in})))$$ (4)

$$x_{out}=FFN(x_{in})+{\hat{x}}_{in}+x_{in}$$ (5)

Here, *x*_*in*_ is the input feature map from the multi-head attention. ${\hat{x}}_{in}$ is the output of Adapter. *x*_*out*_ is the output of AdaptFFN. Our AdaptFFN module is lightweight. And its number of newly introduced parameters is low. The specific features generated by this parallel-designed adapter module can complement the features with in the fixed branch, thus achieving better feature integration. Furthermore, it can provide different scales of semantic information to the encoder and decoder, which enhances the sensory field of the network and thus improves the detection performance of the network.

### 3.4 Loss function

Due to a good loss for bounding box regression should consider three important geometric factors, i.e., overlap area, central point distance and aspect ratio. Therefore, in order to strengthen the constraints on the network localization loss, the regression mechanism for optimizing the bounding box, we use a linear combination of the smooth *L*_1_ loss and the complete IoU loss. The smooth *L*_1_ loss function is formulated as:

$$L_{s1}(x)=\{\begin{array}{c} 0.5x^{2},\;|x|<1,\\ |x|-0.5,|x|\geq 1. \end{array}$$ (6)

Where x denotes the numerical difference of the predicted box and the target box. In the bounding box regression, the smooth *L*_1_ loss function operates on the detection error. Compared with *L*_1_ loss, the smooth *L*_1_ loss is a robust *L*_1_ loss that is less sensitive to outliers than the *L*_2_ loss.

The CIoU loss function is formulated as:

$$L_{CIoU}=1-IOU+\frac{\rho^{2}(b,b^{gt})}{c^{2}}+\alpha \upsilon$$ (7)

$$\upsilon =\frac{4}{\pi^{2}}{\left(arctan\frac{w^{gt}}{h^{gt}}-arctan\frac{w}{h}\right)}^{2}$$ (8)

$$\alpha =\frac{\upsilon }{(1-IoU)+\upsilon }$$ (9)

$$IoU=\frac{b⋂b^{gt}}{b⋃b^{gt}}$$ (10)

Where b and *b*^*gt*^ denote the central points of the predicted box and the target box, *ρ* is the Euclidean distance, and c is the diagonal length of the smallest enclosing box covering the predicted box and the target box. In addition, *α* is a positive trade-off parameter, and *υ* measures the consistency of aspect ratio. Overall, our bounding box loss is defined as

$${L=\lambda }_{s1}L_{s1}+\lambda_{CIoU}L_{CIoU}$$ (11)

Where *λ*_*s*1_ and *λ*_*CIoU*_ are hyperparameters. The linear combination loss of smooth *L*_1_ and CIoU does so by combining the advantages of the two losses, i.e., fully considering the overlap area, center point distance, and aspect ratio of the bounding box, and making the regression of the bounding box more stable.

## 4. Experiments

In this section, we analyze our proposed methods against state-of-the-art object detection algorithms on the RUOD and DUO datasets [43, 44]. In addition, we perform effective ablation experiments, which are described in detail in this section.

### 4.1 Datasets

The experimental dataset uses the RUOD underwater optical image dataset, which contains underwater images of fish, diver, starfish, corals, turtle, echinus, holothurian, scallop, cuttlefish, and jellyfish for a total of 10 categories, as well as the labeling information of the corresponding images. The RUOD dataset has 14,000 images labeled with a total of 74,903 object labels. Among them, the training set contains 9800 images and the validation set contains 4200 images.

And the DUO dataset contains underwater small-scale object images of holothurian, echinus, scallop, and starfish for a total of 4 categories. The total number of objects is 74,515. Holothurian, echinus, scallop, and starfish are 7,887, 50,156, 1,924, and 14,548, respectively. Among them, the training set contains 6671 images and the validation set contains 1111 images.

### 4.2 Experimental details

We follow the DETR training protocol [45]. We train the model on the RUOD and DUO datasets for 20 epochs, with the AdamW optimizer [46]. The learning rate is dropped by a factor of 10 after 10 epochs. The learning rates for the backbone and the transformer are initially set to be 0.00001 and 0.0001, respectively. The weight decay is set to 0.0001. The number of object queries is set to 100. We set the batch size to be 4 to reach a balance. As for the balanced parameters of loss, *λ*_*s*1_ and *λ*_*CIoU*_ are set to 5, 2 respectively. We conduct our experiments on an experimental platform equipped with an AMD EPYC 7543 @ 2.80GHz and a NVIDIA RTX A5000 GPU with 24GB memory. The software environments are CUDA 11.7 and Python 3.8.10.

We use the augmentation scheme same as DETR: resize the input image such that the short side is at least 480 and at most 800 pixels and the long side is at most 1333 pixels; randomly crop the image such that a training image is cropped with a probability 0.5 to a random rectangular patch.

### 4.3 Comparison with other models

To validate the effectiveness of our model, we evaluate it comprehensively on the RUOD dataset and compare it with other state-of-the-art models. To further ensure the fairness of the comparison experiments, we try to set the backbone network to be consistent with that of the original DETR, i.e., ResNet50, or use DarkNet53 with about the same number of parameters as ResNet50. Detailed comparisons are reported in Table 1. We select four categories of nine mainstream networks, the first category is one-stage network represented by SSD, RetinaNet, FreeAnchor, YOLOV3, YOLOX, the second category is two-stage network represented by Faster R-CNN, Cascade R-CNN, the third category is anchor-free network represented by FCOS, and the fourth category is the transformer-based DETR. In particular, we use the original DETR model as our baseline model.

**Table 1:** Comparison between models on RUOD.

Model	Backbone	AP	AP_50_	AP_75_	AP_S_	AP_M_	AP_L_
Faster R-CNN	ResNet50	44.2	77.3	45.8	12.2	34.2	48.9
SSD	VGG16	46.5	76.8	49.1	10.9	32.3	51.5
RetinaNet	ResNet18	48.9	77.4	52.4	16.7	34.5	54.2
Cascade R-CNN	ResNet50	51.8	78.6	56.4	11.8	37.0	57.6
YOLOV3	DarkNet53	43.9	77.5	44.6	8.5	30.4	48.3
FCOS	ResNet50	48.8	78.1	51.7	16.9	36.9	54.0
FreeAnchor	ResNet50	50.9	80.1	54.9	14.6	36.1	56.3
YOLOX	DarkNet53	50.4	78.7	54.2	12.5	36.7	54.8
DETR	ResNet50	53.4	80.8	56.3	12.4	37.6	58.9
Ours	ResNet50	54.7	82.6	57.9	16.9	38.4	60.3

As can be known from Table 1, our proposed model achieves 54.7% *AP*, 82.6% *AP*_50_ and 57.9% *AP*_75_. Our proposed model significantly outperforms state-of-the-art object detection algorithms in all evaluation metrics. In addition, compared with the baseline model, our proposed network improves 1.3 percentage points in *AP*, 1.8 percentage points in *AP*_50_, and 1.6 percentage points in *AP*_75_. More importantly, the performance of our model on small-size underwater images is improved by 4.5 percentage points over the baseline model.

**Fig 4:**
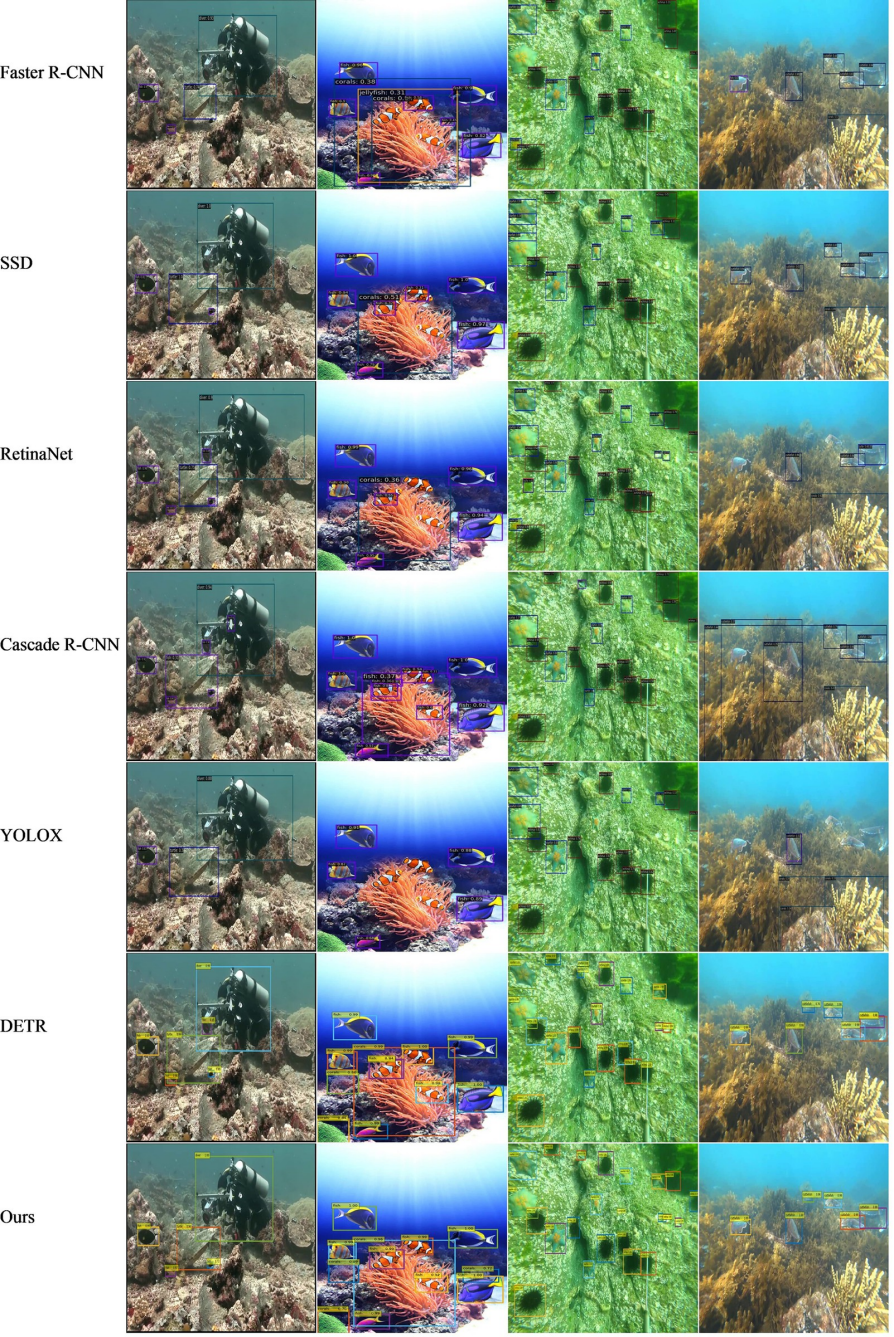
Results of different methods for underwater object detection.

Fig 4 shows the detection of four different images with the best weights obtained from the training of each network, including small underwater and multi-scale objects. In addition, all four images have some noise interference, color distortion, and image blurring, which poses a challenge for correctly identifying the target object and the background class. As can be known from Fig 4, our method has better performance than the other methods. Specifically, RetinaNet, Cascade R-CNN, and the original DETR all incorrectly detect the diver’s camera as a fish when detecting the first image, due to the similarity in color and morphology between the fish and the camera, which causes the detector to make a false detection. When detecting the fish and coral schools in the second image, our model can well recognize the relationship between occlusion and overlap of the two and accurately differentiate them. When detecting the starfish and sea urchin in the third image, the other detectors did not extract the features of the small objects very well, resulting in both being missed to some extent. And our model still has good detection of small objects like starfish and echinus. The cuttlefish in the fourth image is similar in color to the coral colony in the background and has a strong camouflage effect visually, at which point our model can still accurately identify it.

In summary, our improved model has higher accuracy in detecting multiple categories at the same time and in targeting small underwater objects, and it can well reduce the occurrence of missed and false detection. Our proposed learnable query recall mechanism helps the query to efficiently interact with information during the network training process, reduces the interference of image noise, and improves the detection of underwater objects to some extent. Although most of the networks in the comparison experiments use different feature pyramid structures to enrich the feature information during the feature extraction phase, the captured features are still not effective in detecting small objects and multi-scale targets during the deepening of the network structure. And we obtain a richer fine-grained representation by embedding lightweight AdaptFFN modules in each encoder and decoder to enhance the small object signals. Also, the more accurate weighted localization loss further improves the detection accuracy of underwater objects. Next, we will conduct a series of ablation experiments to validate the effectiveness of the proposed method.

### 4.4 Ablation experiments

#### 4.4.1 Effectiveness of each component

In order to validate the effectiveness of the proposed methods, we conduct a series of ablation experiments with the software equipment and algorithmic framework unchanged. The ablation experiments focus on the role of learnable query recall, AdaptFFN, and weighted localization loss function in the network. Table 2 shows the impact of each method on the evaluation metrics when performing ablation experiments.

**Table 2:** Effectiveness of each component of our model.

Learnable Query Recall	AdaptFFN	Weighted Localization Loss	AP	AP_50_	AP_75_	AP_S_	AP_M_	AP_L_
			53.4	80.8	56.3	12.4	37.6	58.9
√			54.3	81.4	57.6	13.7	38.5	59.9
√	√		54.5	81.6	57.8	16.9	38.0	60.1
√		√	54.5	81.4	57.7	14.1	38.9	60.0
√	√	√	54.7	82.6	57.9	16.9	38.4	60.3

As shown in Table 2, when the learnable query recall mechanism is added, *AP*, *AP*_50_, and *AP*_75_ are improved by 0.9%, 0.6%, and 1.3%, respectively, compared with the baseline model. This illustrates how well the learnable query recall mechanism can introduce early supervisory signals for decoder queries and reduce noise interference. The supervisory query signals of different stages are fused with each other, which can effectively help the final query to capture the object and improve the detection accuracy. When learnable query recall mechanism is used in conjunction with AdaptFFN, *AP*, *AP*_50_, *AP*_75_ bring 1.1%, 0.8%, and 1.5% improvement respectively. In particular, we found a significant increase of 4.5% in *AP*_*S*_. This illustrates that AdaptFFN improves the sensory field of the network for the encoding and decoding phases, retains certain fine-grained information, and is able to provide rich multi-scale feature information, which effectively improves the detection accuracy of small underwater objects. When learnable query recall and weighted localization loss function are used together, *AP*, *AP*_50_, *AP*_75_ bring 1.1%, 0.6%, and 1.4% improvement respectively. This is because the weighted localization loss function optimizes the regression of the bounding box and effectively helps the query to localize the prediction box, thus further improving the accuracy of object detection. When learnable query recall mechanism, AdaptFFN and weighted localization loss function are used simultaneously, *AP*, *AP*_50_, *AP*_75_ are improved by 1.3%, 1.8% and 1.6%, respectively. The above ablation experimental results show that the proposed methods are effective and can be well applied to underwater object detection scenarios.

#### 4.4.2 Effectiveness of the middle dimension

We also analyzed the effect of the middle dimension in the AdaptFFN module, as shown in Table 3. The middle dimension not only determines the number of parameters introduced by the AdaptFFN, but also affects the sensory field of the network. When the middle dimension is 32, i.e., the input dimension is down-sampled by a factor of 8, decent performance is achieved, about 53.9% of *AP*. At this point, the number of newly added parameters is 0.42M, which corresponds to 1.02% of the baseline model’s extra parameters. As the middle dimension continues to increase, performance continues to improve. When the middle dimension is equal to 512, the number of parameters introduced is 3.37M, which is equivalent to 7.56% of the extra parameters, and the AP metrics is saturated. While the middle dimension increases to 1024, the AP metric decreases slightly. Therefore, we choose the middle dimension to be 512 for a better tradeoff. A suitable middle dimension allows the branch features generated by the adapter module to effectively complement the features in the original branch in order to achieve better feature fusion.

**Table 3:** Effectiveness of the middle dimension.

mid dim	#params	AP	AP_50_	AP_75_	AP_S_	AP_M_	AP_L_
32(/8)	0.42M	53.9	81.2	56.6	14.5	37.9	59.5
64	0.62M	54.5	82.4	57.8	14.1	38.2	60.1
128	1.01M	54.0	81.2	57.2	15.4	37.9	59.7
256	1.80M	54.5	81.4	57.8	16.5	38.4	**60.4**
512	3.37M	**54.7**	**82.6**	**57.9**	**16.9**	38.4	60.3
1024	6.53M	54.3	81.4	57.3	16.2	**38.7**	60.0

### 4.5 Analysis of small-scale objects on the DUO dataset

In order to better illustrate the performance improvement of the proposed model on small-scale objects, we evaluate the performance metrics on the DUO dataset against the original DETR. The DUO dataset contains four common categories of underwater small objects. Table 4 shows the accuracy of each category and the average accuracy across all categories. As can be known from Table 4, our model outperforms the baseline model in all categories of performance metrics. Furthermore, compared with the baseline model, our proposed network improves 7.6 percentage points in *AP*_*S*_. Therefore, our proposed model can effectively improve the detection performance of underwater objects, especially for small-scale objects.

**Table 4:** Analysis of small-scale objects on the DUO dataset.

Model	Holothurian	Echinus	Scallop	Starfish	AP	AP_S_	AP_M_	AP_L_
DETR	55.3	59.4	39.8	63.1	54.4	40.3	56.4	54.0
Ours	55.9	61.6	41.4	65.4	56.1	47.9	57.4	55.4

## 5. Discussion

Our proposed network integrates a learnable query recall mechanism, a lightweight adapter, and a linear combination loss to enhance the ability of the original DETR to detect underwater objects. The results on the RUOD and DUO datasets validate the effectiveness of the proposed network model. Our model outperforms other mainstream object detection models in all performance metrics. The conducted ablation experiments confirm the effectiveness of the proposed methods. The learnable query recall mechanism can enhance the prediction of underwater target classes and locations with supervised signals from early queries. And the lightweight adapter module uses more comprehensive feature representations, thus enabling the network to identify previously difficult to recognize objects. In particular, it is a plug-and-play module and can significantly improve the recognition accuracy of small-scale objects with a small increase in the number of model parameters. In the future, we will focus on improving the generalization of the model by applying it to unsupervised and multi-modal domains.

## 6. Conclusion

In this paper, we propose a DETR-based underwater object detection network. To mitigate the influence of image noise, we propose a learnable query recall mechanism to improve the accuracy of underwater detection by adding supervisory signals to the query. In order to improve the efficient detection of small and irregular underwater objects, we design a lightweight adapter module. Also, the regression of the bounding box is optimized using the weighted loss with smooth *L*_1_ loss and CIoU loss. Combining these methods, our proposed network outperforms other state-of-the-art object detection algorithms on the RUOD dataset. Comprehensive ablation studies show the effectiveness of the proposed methods. Next, we will conduct further research on unsupervised and multi-modal underwater object detection, expecting to further improve the efficiency of underwater object detection and make some contributions to the industry.
